# Viscoelastic properties of *Pseudomonas aeruginosa* variant biofilms

**DOI:** 10.1038/s41598-018-28009-5

**Published:** 2018-06-26

**Authors:** Erin S. Gloag, Guy K. German, Paul Stoodley, Daniel J. Wozniak

**Affiliations:** 1 Department of Microbial Infection and Immunity, Microbiology, The Ohio State University, Columbus, OH 43210 USA; 2 Department of Biomedical Engineering, Binghamton University, Binghamton, NY 13902 USA; 3 Department of Orthopedics, The Ohio State University, Columbus, OH 43210 USA; 4 National Centre for Advanced Tribology at Southampton, University of Southampton, Southampton, SO17 1BJ UK

**Keywords:** Biophysics, Biofilms

## Abstract

*Pseudomonas aeruginosa* evolves during chronic pulmonary infections of cystic fibrosis (CF) patients, forming pathoadapted variants that are persistent. Mucoid and rugose small-colony variants (RSCVs) are typically isolated from sputum of CF patients. These variants overproduce exopolysaccharides in the biofilm extracellular polymeric substance (EPS). Currently, changes to the biophysical properties of RSCV and mucoid biofilms due to variations in EPS are not well understood. This knowledge may reveal how lung infections resist host clearance mechanisms. Here, we used mechanical indentation and shear rheometry to analyse the viscoelasticity of RSCV and mucoid colony-biofilms compared to their isogenic parent at 2-, 4-, and 6-d. While the viscoelasticity of parental colony-biofilms underwent fluctuating temporal changes, in contrast, RSCV and mucoid colony-biofilms showed a gradual progression to more elastic-solid behaviour. Theoretical indices of mucociliary and cough clearance predict that mature 6-d parental and RSCV biofilms may show reduced cough clearance from the lung, while early mucoid biofilms may show reduced clearance by both mechanisms. We propose that viscoelasticity be considered a virulence property of biofilms.

## Introduction

In the cystic fibrosis (CF) lung mucus accumulates due to impaired clearance forming a niche that is readily colonised by bacteria. Infecting organisms form biofilms within the mucus lining^1,2^. This results in chronic pulmonary infections that are responsible for the morbidity and mortality of CF patients^3^, with *Pseudomonas aeruginosa* one of the responsible pathogens^4^. Compounding the chronicity of these infections is the ability of *P*. *aeruginosa* to pathoadapt, resulting in the evolution and selection of variants that are more fit. Emergence of these variants is associated with increased treatment difficulties and worsening patient prognosis^5^.

Pathoadaptation is the process where microorganisms adapt to a new pathogenic niche by acquiring genetic mutations. These adaptations result in diversification of the population through the emergence of variants that are more fit in the given pathogenic niche^6^. Two unique classes of pathoadapted variants isolated from the lungs of CF patients that we are interested in are mucoid and rugose small-colony variants (RSCVs)^7,8^. These variants show phenotypic differences compared to the isogenic parental strain due to overproduction of different exopolysaccharides in the biofilm extracellular polymeric substance (EPS). Mucoid variants overproduce alginate^7^, which is a negatively charged acetylated polymer of guluronic and mannuronic acid^9^. RSCVs overproduce both Psl (Polysaccharide synthesis locus) and Pel (Pellicle polysaccharide)^8,10^. Psl is a neutrally charged polymer of mannose, rhamnose and glucose monosaccharides, while Pel is a positively charged polymer of acetylgalactosamine and acetylglucosamine^11–14^. Physiological benefits in an infection, such as hyper-biofilm formation^10,15^, increased tolerance to antimicrobials^16^ and evasion of the host immune system^17,18^ have been well documented for these variants. In recent years biofilm viscoelasticity has also been implicated in facilitating bacterial persistence during an infection^19^. How changes to the EPS of *P*. *aeruginosa* biofilms influences biofilm viscoelasticity is a growing area in the field of biofilm research. Furthermore it is unclear if changes in biofilm viscoelasticity afford any advantages to the community in an infection.

In contrast, changes in mucus viscoelasticity clearly impact the level of clearance from the lung, by both mucociliary and cough mechanisms. In healthy individuals mucociliary clearance is the primary mechanism, whereby cycles of cilia beating drive mucus to the pharynx^20^. In diseased lung states, such as CF, mucus hypersecretion and changes in mucus viscoelasticity impair mucociliary clearance. For these conditions, mucus is predominately expelled by cough clearance^20^, where a burst of high-velocity air drives mucus into the larger airways^21^.

Theoretical mucociliary- and cough-clearance indices (MCI and CCI respectively) have been determined for sputum (expectorated mucus), derived from *in vitro* lung clearance models^22,23^. These indices correlate sputum viscoelasticity to predicted levels of clearance from the lung. The MCI predicts that mucus elasticity correlates with mucociliary clearance^22^. Prior studies proposed that mucus elasticity promotes the transfer of energy from cilia, while increased viscosity prevents the movement of cilia through mucus^24^. In contrast, the CCI predicts that mucus viscosity correlates with cough clearance^25^. It has been proposed that high viscosity allows mucus to remain intact, while low elasticity promotes airflow-mucus interactions by preventing mucus recoil during the burst of high-velocity air^24^. Therefore mucus viscoelasticity typically favours clearance by one mechanism, as properties that promote mucociliary clearance inhibit cough clearance and visa versa. This indicates that the viscoelasticity of healthy mucus maintains a balance that promotes clearance by both mechanisms^24,25^.

We predict that the viscoelasticity of biofilms, similar to mucus, influence their mechanical removal from the lung during infection. Here, we used mechanical indentation and shear rheometry to determine how the biophysical properties of *P*. *aeruginosa* wildtype (WT), mucoid, and RSCV biofilms change overtime and implicate biofilm viscoelasticity to theoretical mucociliary and cough clearance from the lung. We propose that viscoelasticity be included in the virulence factor properties that biofilms possess.

## Results

### *P*. *aeruginosa* colony-biofilms

To determine if the viscoelasticity of *P*. *aeruginosa* pathoadapted variants differ from the parent strain due to changes to the EPS, mechanical tests were performed on representative RSCV and mucoid colony-biofilms and compared to the isogenic parent WT. To determine how the mechanical properties change over time 2-, 4-, and 6-d colony-biofilms were analysed using indentation or shear rheometry.

**Figure 1:**
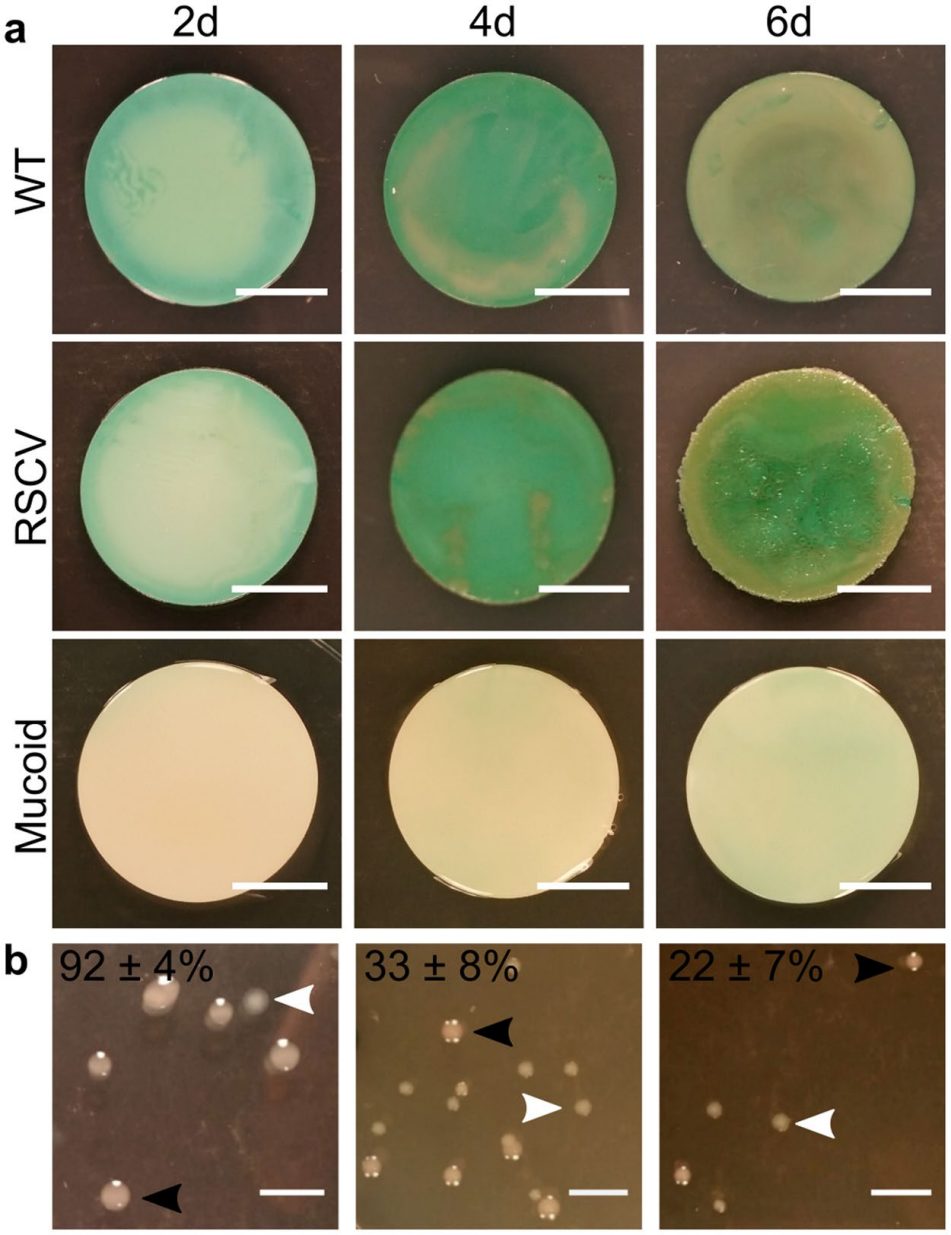
*P*. *aeruginosa* colony-biofilms. Images of (**a**) WT, RSCV and mucoid *P*. *aeruginosa* colony-biofilms (labelled) at 2-, 4- and 6-d (left, middle and right panel respectively). Scale bar 10 mm. (**b**) Mucoid (black arrowhead) and non-mucoid revertant (white arrowhead) populations isolated from 2-, 4-, 6-d mucoid colony-biofilms (left, middle and right panel respectively). Frequency of mucoid colonies are indicated in the top left had corner as a percentage of total colonies. Mean ± SD, n = 3. Scale bar 5 mm.

From macroscopic observations colony-biofilm development was evident over the 6 days (Fig. 1). Green pigment production, indicative of pyocyanin production, by WT biofilms increased from 2-d through to 6-d (Fig. 1a). RSCV colony-biofilms were macroscopically similar to WT on 2-d. However by 4-d, wrinkling was apparent around the periphery of the biofilm, with this pattern extending across the whole biofilm by 6-d (Fig. 1a). This biofilm morphology is equivalent to the rugose phenotype of RSCV colonies grown directly on agar^8,10^. No pigmentation was observed for the 2-d mucoid colony-biofilms (Fig. 1a), consistent with previous reports that found mucoidy suppresses pyocyanin production^26^. On 4-d and 6-d increasing non-mucoid reverted sub-populations, which correlated to increasing levels of pigmentation at those timepoints, were isolated from mucoid colony-biofilms (Fig. 1b). Again this is consistent with previous reports that observed pyocyanin production returned to wildtype levels in mucoid strains that developed a secondary suppressor mutation^26^.

### Uniaxial indentation measurements reveal that mucoid colony-biofilms are more flexible than WT

**Figure 2:**
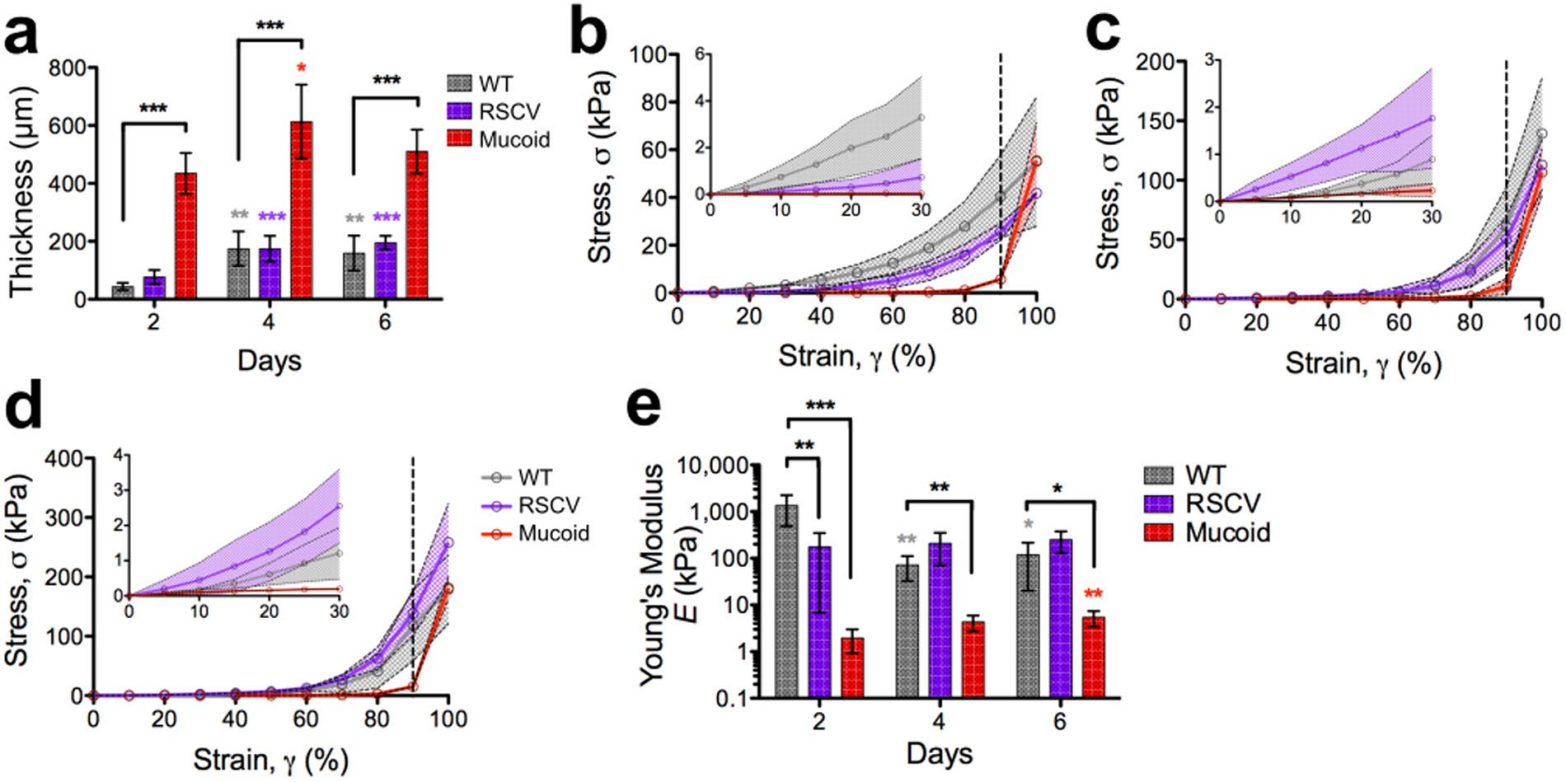
Mucoid colony-biofilms are softer than WT. (**a**) Thicknesses of colony-biofilms determined from the uniaxial compression measurement. Stress-strain curves of (**b**) 2-d, (**c**) 4-d and (**d**) 6-d *P*. *aeruginosa* colony-biofilms from uniaxial compression measurements. The dotted black line at 90% strain (γ) indicates where the underling filter likely influenced the data. The inset depicts the region of the curve from 0–30% strain (γ). Legend depicted in (**d**) is equivalent for (**b**) and (**c**). (**e**) Young’s modulus of the lower linear portion of the force-displacement curve, corresponding to 0–30% strain (γ). Data presented as mean ± SD; n = 6. Black * indicates comparisons depicted by the line. Coloured * indicates comparisons to 2-d of the given biofilm. *p-value < 0.05, **p-value < 0.01, ***p-value < 0.001.

Uniaxial compression measurements were performed to determine the stiffness of *P*. *aeruginosa* colony-biofilms under a normal force (i.e. perpendicular to the growth surface). For these measurements the probe was lowered onto the biofilm and the force required to compress the biofilm recorded. From this analysis, colony-biofilm thickness was also determined (Fig. 2a). The thickness of mucoid biofilms remained relatively constant across the timepoints (approximately 500 μm) and was significantly greater than WT biofilms (Fig. 2a). The thickness of WT and RSCV colony-biofilms were comparable at each timepoint (Fig. 2a). However by 4-d the biofilm thicknesses increased significantly to approximately 200 μm. This thickness was maintained thereafter (Fig. 2a).

The stress-strain curves showed J-shaped relationships for all biofilms, indicating that as the biofilms were compressed they became increasingly stiffer (Fig. 2b–d). However, we anticipate that at strains >90% the underlying filter may have influenced the measurement. This is particularly evident from the sharp increase in the curve of the mucoid biofilm after this point (Fig. 2b–d; dotted black line). The J-shaped response is commonly seen in biological materials, such as skin, and suggests that biofilm EPS polymers progressively align with increasing stresses, in the direction of the applied stress^27^. This response has previously been observed for *Streptococcus mutans* hydrated static biofilms^28^ and mixed biofilms of *P*. *aeruginosa*, *P*. *fluorescens*, *Klebsiella pneumoniae* and *Stenotrophomonas maltophilia*^29^.

To quantify colony-biofilm stiffness, the Young’s modulus was determined from the low linear regime of the force-displacement curve within the 0–30% strain region (Fig. 2b–d; inset) using equation (1). WT colony-biofilms were stiffer on 2-d compared to 4-d and 6-d (Fig. 2e). The Young’s modulus for RSCV colony-biofilms was consistent across the three timepoints (Fig. 2e), while mucoid biofilms became stiffer from 2-d to 6-d (Fig. 2e). Across all timepoints WT colony-biofilms were stiffer compared to mucoid (Fig. 2e). However, WT biofilms were only stiffer than RSCV on 2-d (Fig. 2e).

**Figure 3:**
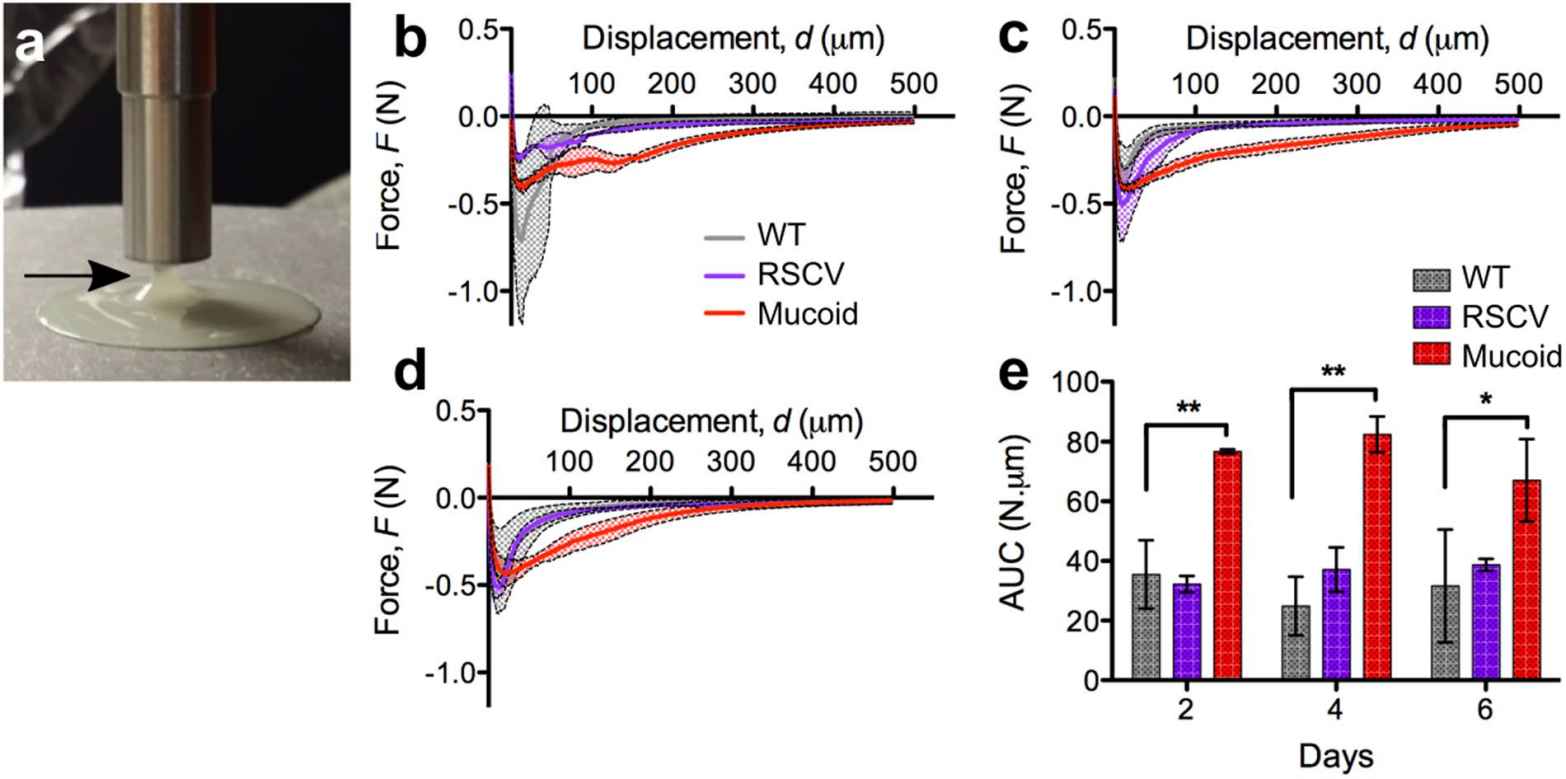
Mucoid colony-biofilms are more cohesive. (**a**) 2-d mucoid colony-biofilm after being compressed with 8 mm probe. Arrow indicates the biofilm adhered to the probe as it is raised off the biofilm. Force-displacement curves of (**b**) 2-d (**c**) 4-d and (**d**) 6-d *P*. *aeruginosa* colony-biofilms from the unloading phase of the squeeze-pull off measurements. In (**d**) the mean line for WT is underneath that of the RSCV. The negative force corresponds to the force exerted by the biofilm on the probe as it is retracted. The x-axis is the distance that the probe was raised out of the biofilm, with 0μm set to where compression of the biofilm (squeeze) stopped. Legend depicted in (**c**) is equivalent for (**b**) and (**d**). Data presented as mean ± SD; n = 4. (**e**) Area under the curve (AUC) of the curves depicted in (**b–d**). Data presented as mean ± SD, n = 4. *p-value < 0.01, **p-value < 0.001.

As the probe was raised at the end of these measurements it was observed that the biofilm remained attached, forming fibril structures before detaching (Fig. 3a; black arrow). Therefore, squeeze-pull off measurements were performed to measure the adhesion of *P*. *aeruginosa* colony-biofilms. For these measurements the biofilms were compressed as above, after which the force required to raise the probe off the biofilm was measured. All biofilms displayed some degree of adhesion to the probe, as indicated by the negative force (Fig. 3b–d), demonstrating that the biofilm was pulling back as the probe was retracted. The area under the curve (force x distance), representing the work done by the probe to rise off and stretch the biofilm to its breaking strain was determined. This revealed that mucoid colony-biofilms were more cohesive compared to WT at all timepoints (Fig. 3e). Cohesion of WT and RSCV colony-biofilms were comparative. However, individual *P*. *aeruginosa* biofilms did not change over the analysed time period (Fig. 3e).

### Oscillatory stress sweeps reveal that mature mucoid colony-biofilms have a higher yield stress compared to WT

**Figure 4:**
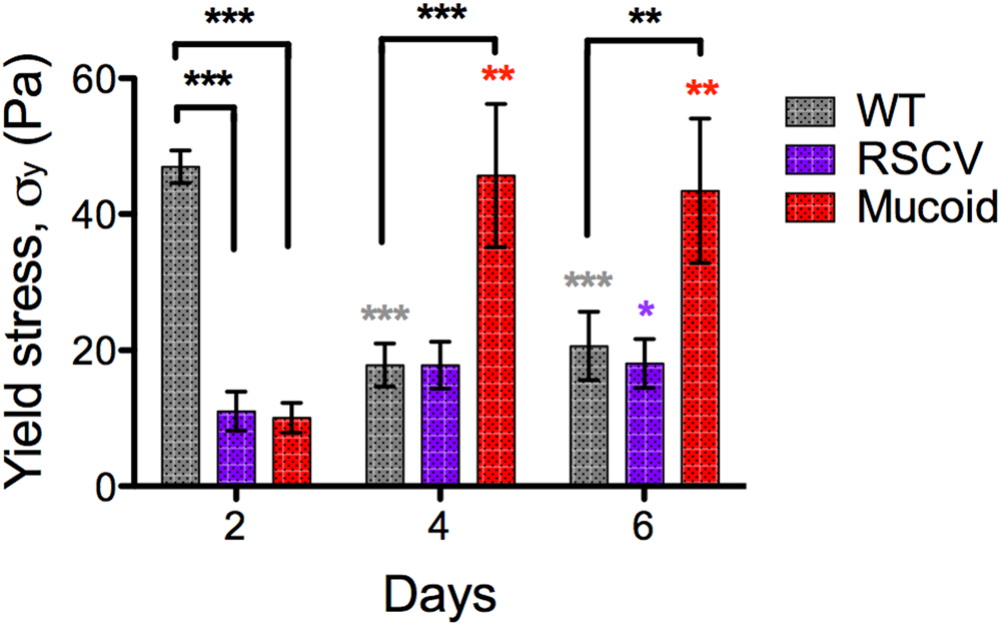
Yield stress of *P*. *aeruginosa* colony-biofilms. Stress sweeps were performed on WT, RSCV and mucoid colony biofilms at 2-, 4- and 6-d by incrementing the stress from 0.01–100 Pa (Supplementary Fig. S1). The yield stress (σ_y_) was taken to be the point of viscoelastic crossover where the storage modulus (G′) and the loss modulus (G″) intersected in Supplementary Fig. S1. Data presented as mean ± SD, n = 4. Black * indicates comparisons depicted by the line. Coloured * indicates comparisons to 2-d of the given biofilm. *p-value < 0.05, **p-value < 0.01, ***p-value < 0.001.

To determine the strength of *P*. *aeruginosa* colony-biofilms when exposed to a shear force (i.e. parallel to the growth surface), oscillatory stress sweeps were performed (Supplementary Fig. S1). For this measurement the shear stress was incremented and the elastic and viscous responses of the biofilm recorded as the storage (G’) and loss (G”) moduli respectively (See Supplementary Methods, Supplementary Fig. S1). The point where G’ and G” became equal was used as an indicator of the yield stress (Fig. 4)^30^. This is the stress where the biofilm transitions from being more solid-like to more liquid-like. This measurement was used here as a surrogate indicator of strength. WT colony-biofilms were stronger on 2-d compared to 4-d and 6-d (Fig. 4). This temporal behaviour of WT biofilms reflects the changes observed for the Young’s modulus (Fig. 2e). Furthermore, on 2-d, WT colony-biofilms were stronger compared to RSCV and mucoid biofilms (Fig. 4). Interestingly, 4-d mucoid colony-biofilms were significantly stronger compared to mucoid 2-d biofilms (Fig. 4), with the yield stress being consistent across to 6-d (Fig. 4). Mucoid colony-biofilms on 4-d and 6-d were also stronger compared to WT (Fig. 4). The strength of RSCV colony-biofilms slowly increased across the analysed time points (Fig. 4). However, the yield stress for RSCV and WT colony-biofilms on 4-d and 6-d were comparative (Fig. 4).

### Shear creep-recovery measurements reveals that mature RSCV and mucoid biofilms are less viscous and stiff compared to WT biofilms

**Figure 5:**
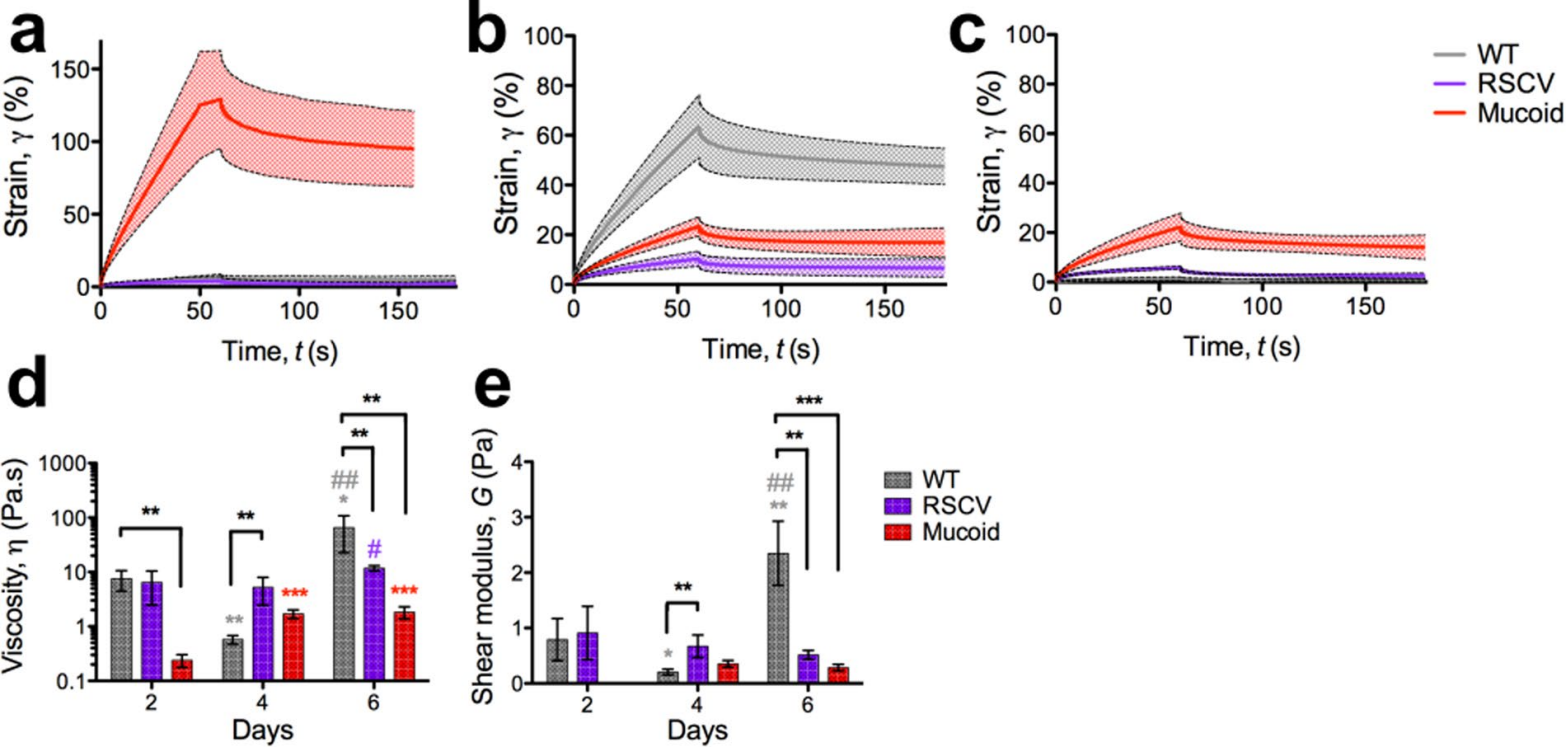
Mature RSCV and mucoid colony-biofilms are less viscous and stiff compared to WT. Creep-recovery measurements were performed on (**a**) 2-d, (**b**) 4-d and (**c**) 6-d *P*. *aeruginosa* colony-biofilms. From the creep-recovery curves the (**d**) viscosity (η) and (**e**) shear modulus (*G*) was determined. Data presented as mean ± SD, n = 4. Black * indicates comparisons depicted by the line. Coloured * indicates comparisons to 2-d of the given biofilm. *p-value < 0.05, **p-value < 0.01, ***p-value < 0.001. Coloured # indicates comparison to 4-d of the given biofilm. ^#^p-value < 0.05, ^##^p-value < 0.001.

Static creep-recovery measurements were performed to determine the viscoelasticity of *P*. *aeruginosa* under pseudo-steady state conditions (Fig. 5a–c). For these measurements biofilm deformation was measured as a function of time under an applied shear stress. The stress was then removed and biofilm recovery measured. From these measurements the shear modulus (*G*) (as an indicator of biofilm stiffness under a shear force) and dynamic viscosity (η) of the colony-biofilms were quantified (Fig. 5d,e). The viscoelasticity of WT biofilms showed temporal changes, where on 2-d WT colony-biofilms showed little deformation (Fig. 5a). In contrast, on 4-d WT colony-biofilms were more readily deformed (Fig. 5b) and were less viscous and stiff (Fig. 5d,e). On 6-d WT colony-biofilms returned to a stiffer phenotype, however were more viscous and rigid compared to 2-d (Fig. 5d,e).

In comparison, the viscoelastic response of RSCV biofilms remained relatively steady over time (Fig. 5), except for a slight increase in viscosity from 4-d to 6-d (Fig. 5d). On 2-d, WT and RSCV colony-biofilms were similar. However, on 4-d and 6-d the biophysical comparisons between WT and RSCV biofilms changed due to the temporal changes of WT biofilms. On 4-d, when WT biofilms were softer, RSCV biofilms were more viscous and stiff in comparison (Fig. 5d,e). While on 6-d, when WT biofilms became more solid, RSCV biofilms were less viscous and stiff in comparison (Fig. 5d,e).

As their observed colony-biofilm morphology would suggest (Fig. 1a), 2-d mucoid colony-biofilms were dominated by viscous behaviour and exhibited flow upon initial recovery (Fig. 5a); therefore no elastic recovery could be measured to determine the shear modulus (Fig. 5d). WT colony-biofilms were more viscous compared to mucoid at this timepoint (Fig. 5e). On 4-d and 6-d the viscoelastic response of mucoid colony-biofilms were similar, showing reduced deformation compared to 2-d (Fig. 5a–c). This correlated to an increased viscosity (Fig. 5d) and shear modulus (Fig. 5e). Compared to WT colony-biofilms, on 4-d the viscosity and shear modulus were similar, however on 6-d WT biofilms were more viscous and stiffer (Fig. 5d,e).

### Oscillatory frequency sweeps reveals changes in WT, RSCV and mucoid biofilm viscoelasticity across dynamic ranges

**Figure 6:**
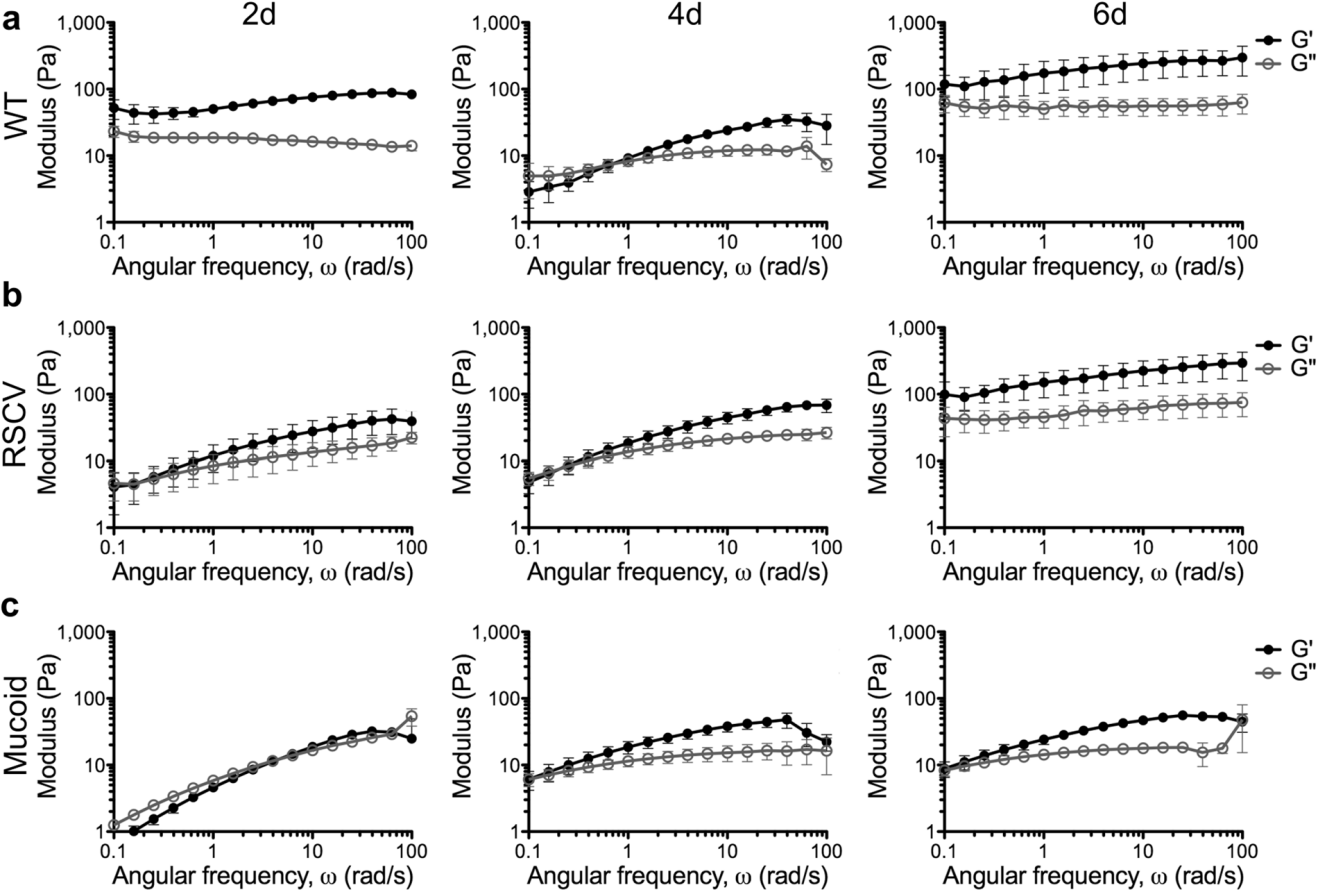
Frequency sweeps of *P*. *aeruginosa* colony-biofilms. Frequency sweeps were performed on (**a**) WT, (**b**) RSCV and (**c**) mucoid colony biofilms at 2-, 4- and 6-d (labelled) by incrementing the angular frequency (ω) from 0.1–100 rad/s. Data presented as mean ± SD; n = 4.

To measure how the complex viscoelastic parameters of *P*. *aeruginosa* colony-biofilms varied as a function of frequency, dynamic oscillatory frequency sweeps were performed (Fig. 6). This analysis shows how a material will behave when subjected to an oscillating force at any given frequency within the testing range (0.1–100 rad/s).

For WT, 2-d, and 6-d colony-biofilms, both moduli were relatively independent of frequency, with the storage modulus greater than the loss modulus across all frequencies (Fig. 6a). The magnitude of the moduli was greater for 6-d colony-biofilms compared to 2-d, suggesting that WT colony-biofilms displayed more elastic-solid behaviour on 6-d (Fig. 6a). In comparison, WT biofilms on 4-d showed a viscoelastic crossover of the storage and loss modulus, where at high frequencies the biofilm elastic behaviour dominated (G’ > G”) and at low frequencies the viscous behaviour dominated (G” > G’) (Fig. 6a). This fluctuating behaviour is reflective of that observed for the creep-recovery measurements (Fig. 5).

RSCV colony-biofilms showed a gradual progression to more elastic-solid properties over time. 2-d and 4-d biofilms displayed a viscoelastic crossover of the storage and loss modulus (Fig. 6b), similar to WT 4-d biofilms. While, for 6-d RSCV colony-biofilms both moduli were independent of frequency with the storage modulus greater than the loss modulus across all frequencies, similar to WT 6-d biofilms (Fig. 6b). This indicates that 6-d RSCV biofilms displayed more elastic-solid properties compared to 2-d and 4-d (Fig. 6b).

As expected, 2-d mucoid colony-biofilms exhibited viscoelastic fluid behaviour, as the storage and loss moduli were comparative (Fig. 6c). On 4-d and 6-d mucoid colony-biofilms displayed increasing elastic behaviour at higher frequencies, with the storage modulus being dominant at these frequencies (Fig. 6c). Mucoid colony-biofilms were generally more fluid than WT across all three timepoints, with the exception of 4-d where both biofilms showed similar behaviour (Fig. 6a,c).

### Correlation of colony-biofilm viscoelasticity to theoretical mucociliary and cough clearance indices

**Figure 7:**
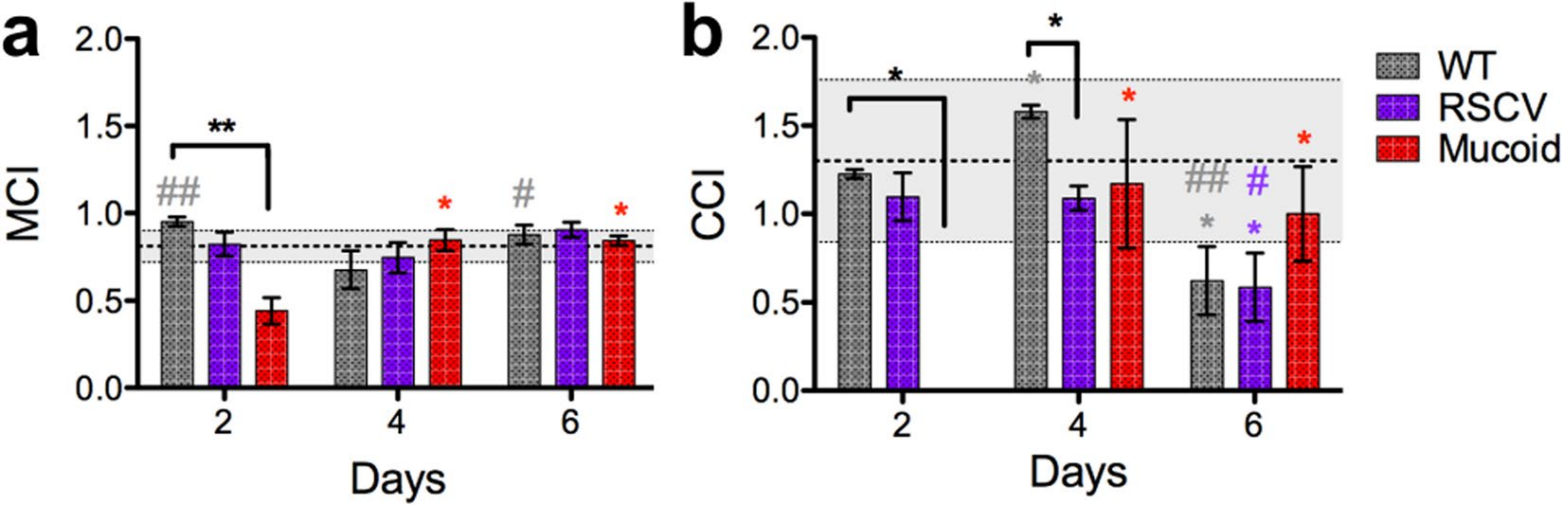
Mucoid colony-biofilms show reduced mucociliary and cough clearance indices. (**a**) The MCI and (**b**) the CCI of *P*. *aeruginosa* colony-biofilms was determined according to equations (2) and (3). The lines at (**a**) 0.81 ± 0.09 and (**b**) 1.3 ± 0.46 indicate the MCI and CCI of CF sputum respectively determined^31^. Data presented as mean ± SD, n = 4. Black * indicates comparisons depicted by the line. Coloured * indicates comparisons to 2-d of the given biofilm. *p-value < 0.05, **p-value < 0.01. Coloured ^#^ indicates comparison to 4-d of the given biofilm. ^#^p-value < 0.05, ^##^p-value < 0.01.

To correlate the viscoelasticity of *P*. *aeruginosa* biofilms determined here to the predicted mechanical clearance from the CF lung, we calculated the mucociliary clearance index (MCI) and the cough clearance index (CCI) (Fig. 7). Here, a higher index indicates a higher theoretical clearance. The MCI and CCI were calculated at an angular frequency of 1 rad/s and 100 rad/s according to equations (2) and (3) respectively. The MCI and CCI of *P*. *aeruginosa* colony-biofilms were similar to those previously reported for CF sputum (Fig. 7; dotted lines)^31^.

2-d WT colony-biofilms showed an intermediate MCI and CCI compared to other timepoints (Fig. 7). The more fluid 4-d WT colony-biofilms had a reduced MCI compared to both 2-d and 6-d (Fig. 7a). However, this correlated to an increased CCI (Fig. 7b). The highly elastic 6-d WT biofilms had a MCI similar to 2-d (Fig. 7a). However, the CCI was reduced compared to both the 2-d and 4-d biofilms (Fig. 7b).

RSCV biofilms on 2-d and 4-d had a similar MCI and CCI (Fig. 7). However, an increase in elastic-solid behaviour of 6-d biofilms correlated to a reduced CCI compared to 2-d and 4-d, similar to WT (Fig. 7b). For the most part the MCI and CCI of RSCV biofilms were equivalent to WT, with the exception of 4-d where WT had an increased CCI (Fig. 7b).

The fluid behaviour of mucoid biofilms on 2-d correlated to a reduced MCI and CCI compared to WT (Fig. 7). Development of partial elastic-solid behaviour of mucoid biofilms on 4-d and 6-d resulted in an increased MCI and CCI compared to 2-d (Fig. 7). At these later timepoints the MCI and CCI of mucoid biofilms were equivalent to WT (Fig. 7).

## Discussion

Here we determined the rheological properties of representative pathoadapted *P*. *aeruginosa* variants compared to their isogenic WT parent. The viscoelasticity of WT colony-biofilms showed extensive temporal changes during biofilm development. 2-d and 6-d WT biofilms showed rheological behaviour reflective of viscoelastic gels, whereas 4-d biofilms behaved as a viscoelastic liquid (Fig. 6 ^32^). This suggests that for 2-d and 6-d biofilms there are higher affinity interactions between EPS components, compared to 4-d. It has recently been proposed that during *P*. *aeruginosa* hydrated biofilm formation, the exopolysaccharide Psl is dominant at early stages, forming a stiff matrix, however Pel exopolysaccharide increases as the biofilm matures, remodeling the matrix and forming a more viscous or malleable structure^33^. This mirrors our observations in the mechanical transitions of 2-d to 4-d WT biofilms. Together with our data this suggests that there might be waves of remodeling or temporal release of different amounts, types or binding affinities of EPS components. However changes in cell density at the time-points analysed may also be having an effect. These observations warrant further investigation to determine if this cyclic behaviour extends across timepoints after 6 days. This will be the focus of future work.

RSCV colony-biofilms showed a gradual progression to more elastic-solid behaviour, where 2-d and 4-d biofilms behaved as viscoelastic liquids and 6-d biofilm as a viscoelastic gel (Fig. 6;^32^). This suggests that over time the EPS network becomes more stable. Interestingly, the viscoelasticity of RSCV and WT colony-biofilms were relatively similar. We had predicted that RSCV biofilms would be stiffer compared to WT, due to the different biofilm morphologies (Fig. 1a). Kovach *et al*. reported that RSCV scraped lawn-biofilms were tougher and more elastic compared to the parent strain^34^. In our hands, scraped lawn-biofilms had a higher yield stress and more elastic-dominate behaviour compared to colony-biofilms (See Supplementary Results, Supplementary Figs S2 and S3). Despite this, there were no significant differences between WT and RSCV scraped lawn-biofilms (Supplementary Figs S2 and S3), similar to what we observed for the colony-biofilms.

The similarities between WT and RSCV biofilms can be explained by considering RSCV colony-biofilms as a two-layered system (See Supplementary Results). This predicts that RSCV biofilms develop a stiff-elastic skin, which encases a softer and more viscous liquid core as the biofilm matures^35,36^. This model would also account for the wrinkled colony-biofilm morphology. The bulk RSCV rheological properties would be influenced by both layers, accounting for the similarities between RSCV and WT biofilms (See Supplementary Results).

Early mucoid colony-biofilms behaved as viscoelastic liquids, presumably due to the hygroscopic nature of alginate^37^. Our observations of the viscoelasticity of mucoid biofilms are in agreement with those identified previously^34,38,39^, validating our findings. Korstgens *et al*. reported that an environmental mucoid strain behaved as a viscoelastic liquid with a Young’s modulus of 6.5 ± 0.5 kPa^38^, approximately what we observed. Furthermore, Wloka *et al*. observed viscoelastic behaviour of two different *P*. *aeruginosa* mucoid biofilms (SG81 and FRD1)^39^ similar to the mucoid biofilms analysed here (PDO300 strain). This suggests that the bulk biophysical properties of mucoid biofilms are conserved despite the originating ancestor strain background. However, biofilms of mucoid mutants with different mannuronic:guluronic acid ratios (M:G) and levels of acetylation have altered viscoelastic properties compared to the parent mucoid biofilms^39^.

We hypothesize that the development of partial elastic-solid behaviour by mucoid colony-biofilms on 4-d and 6-d is due to the increasing presence of reverted non-mucoid populations within the biofilm (Fig. 1b). Reversion to non-mucoid phenotypes is commonly observed for mucoid strains, both *in vitro* and *in vivo*, whereby the cells develop a secondary suppressor mutation^26,40^. On 4-d and 6-d the remaining mucoid population were a minority in the colony-biofilm (Fig. 1b). This suggests that small *P*. *aeruginosa* mucoid populations are sufficient to significantly alter the bulk rheological properties of the biofilm. Furthermore, we observed that later mucoid colony-biofilms were stronger (higher yield stress) than early mucoid and WT biofilms (Fig. 4). This may indicate rheological co-operation of the two populations to form biofilms that exhibit both fluid properties and yielding to mechanical stresses without detaching. This may have important consequences in infection where mixed *P*. *aeruginosa* mucoid and reverted non-mucoid populations are routinely encountered^41^.

In the context of *P*. *aeruginosa* persistence during CF lung infections, previous studies have focused on the diversity of pathoadaptive variants and implications of the adaptive phenotypes to infection^42^. However, changes in biofilm viscoelasticity will also likely have important consequences to *P*. *aeruginosa* persistence in the CF lung, which are yet to be realised.

Recent studies have identified that the biophysical properties of dental biofilms influence their mechanical removal from surfaces and may have significant impacts on oral hygiene^28,43^. However, similar considerations have not been made regarding the mechanical clearance of biofilms from the lung during infection. Our observations here suggest that during chronic pulmonary CF infections, where *P*. *aeruginosa* biofilms and pathoadapted variants are well established, mucociliary and cough clearance may be further compromised by contributions of *P*. *aeruginosa* biofilms’ viscoelastic properties. In support of this hypothesis, a previous study identified that CF patients colonised with *P*. *aeruginosa* had a reduced mucociliary clearance compared to CF patients lacking *P*. *aeruginosa* and healthy control individuals^44^. A separate study observed that cough clearance was reduced in *P*. *aeruginosa* positive children with CF compared with *P*. *aeruginosa* negative CF children^45^. However, our study is the first indication that the viscoelasticity of *P*. *aeruginosa* biofilms may influence clearance from the lung.

Within the lower airways *P*. *aeruginosa* establishes segregated distinct communities^46^. Remodeling or changes in biofilm EPS overtime, as observed here for the WT biofilms, may ensure different levels of clearance of these isolated communities, in the absence of evolved variants. This may be a mechanism to ensure maintenance of some degree, promoting persistence within the lung.

Early mucoid biofilms, or single mucoid populations may contribute to the inhibition of both mucus clearance mechanisms in CF lungs, as predicted by the low MCI and CCI (Fig. 7). However emergence of non-mucoid populations and the development of partial elastic behaviour at later timepoints resulted in increased MCI and CCI (Fig. 7). Interestingly, the cohesiveness of mucoid biofilms did not change over time, despite these populations (Fig. 3e). Compared to healthy mucus, CF mucus has a greater adhesivity which impairs cough clearance^47^. Therefore, despite the higher MCI and CCI of mucoid biofilms at these later timepoints the sticky mucoid EPS may still contribute to the mucus and reduce clearance.

While we realise that the colony-biofilm model analysed here may not directly mimic biofilms in CF pulmonary infections, this experimental design allowed us to easily and reproducibly measure the mechanical properties of *P*. *aeruginosa* biofilms, setting the foundation for our hypothesis proposed here. However, we are currently developing assays to explore these concepts in models that more closely resemble the CF lung environment. These include analysing biofilms grown in artificial sputum media and in tissue culture of CF lung epithelial cells and will be the focus of future work.

Of further interest would be exploring whether therapies routinely used by CF patients alter the mechanical properties of bacterial biofilms. Mucolytics, such as recombinant human DNase (rhDNase; Pulmozyme) and hypertonic saline (7% NaCl), are administered to CF patients in an attempt to loosen the mucus and aid in its mechanical clearance. Human extracellular DNA (eDNA) is a major component of CF mucus and it has been shown that by degrading this eDNA, rhDNase reduces the viscoelasticity of CF sputum^31,48^. eDNA is also a major component of the EPS of bacterial biofilms, including those of *P*. *aeruginosa*, and incubation with DNaseI disrupts the biofilm^49,50^. However, it is yet to be shown if rhDNase influences the mechanical properties of biofilms within CF mucus. Hypertonic saline reduces the viscoelasticity of CF sputum, presumably by re-hydrating the sputum^48^. However, incubating *P*. *aeruginosa* biofilms with NaCl concentrations similar to hypertonic saline had no effect on the mechanical properties, compared to untreated controls^51^. A systematic analysis of how CF therapies, such as mucolytics and antibiotics, impact the mechanical properties of bacterial biofilms has not been performed. In line with what we are proposing here, that the viscoelasticity of biofilms contribute to the tenacity of these infections, this analysis is clearly warranted.

Our data suggests that by evolving variants with different biophysical properties, *P*. *aeruginosa* can insure against mucociliary and cough clearance from the CF lung as well as developing viscoelastic properties, which may facilitate the colonisation of the lung. This adds to the ‘insurance hypothesis’ previously proposed for the evolution of *P*. *aeruginosa* variants during infection^42^ by suggesting further adaptive phenotypes of *P*. *aeruginosa* variants. In agreement with Peterson *et al*.^19^ we propose that the viscoelasticity of the biofilm be included in the arsenal of virulence properties that *P*. *aeruginosa* biofilms possess.

## Materials and Methods

### Colony-biofilms

Bacterial strains used in this study were *P*. *aeruginosa* PAO1 (WT) and isogenic PAO1Δ*wspF* (RSCV; JJH356) and PAO1*mucA22* (mucoid; PDO300^52^). These isogenic mutants were used in this study as representative RSCV and mucoid variants that are clinically derived from CF lung infections^7,8,10^. Overnight cultures were diluted to OD_600nm_ 0.1 into fresh media. Sterile nitrocellulose filter membranes (25 mm, 0.45μm pore size; Milliopore) were floated on diluted culture and transferred onto *Pseudomonas* isolation agar (PIA) (45 g/L PIA powder (BD), 20 mL glycerol), culture side up and incubated at 37 °C. Colony-biofilms were transferred to a new PIA plate every 24 h (See Supplementary Methods).

### Rheometry analysis

#### Rheometer apparatus

A TA Instruments Discovery Hybrid Rheometer-2 (HR-2) with the Peltier plate connected to a heat exchanger (TA Instruments) was used for all rheological measurements (See Supplemental Methods).

#### Uniaxial compression measurements

Indentation measurements were performed using the 8 mm Smart Swap geometry with an approach rate of 1 μm/s. 3 colony-biofilms were analysed at each timepoint, with 2 measurements per biofilm. Contact with the biofilm was determined where the force began to increase after the point of pull-on adhesion (Supplementary Fig. S4). This point was taken to be the thickness of the biofilm^53^.

Force-displacement curves are presented as stress-strain curves. Force (*F*) was converted to stress (σ) by dividing by the area of the geometry (σ = F/π*r*^2^). Displacement was converted to strain (γ) by dividing displacement (Δ*L*) by the thickness (*L*) of the biofilm (γ = Δ*L*/*L*). From the force-displacement curve the Young’s modulus (*E*) was calculated using the force-displacement relationship previously described^54^:

1 $$E=\frac{slope\cdot (1-{v}^{2})}{2r}$$

where the slope is of the force-displacement curve (N/m), *r* is the radius of the probe (*r* = 0.004 m) and *v* is the assumed Poisson’s ratio of a biofilm (*v* = 0.5)^28^. The slope of the force-displacement curve was measured at the region corresponding to 0–30% strain where R^2^ > 0.95.

Squeeze-pull off measurements were performed using the 25mm-sand blasted Smart Swap geometry. The probe was lowered onto the biofilm with an approach rate of 1μm/s until an axial force of 0.5 N was detected (Squeeze). The probe was then raised off the biofilm at the same rate for a distance of 500 μm (Pull off). 1 measurement was performed per colony-biofilm. 4 colony-biofilms at each timepoint were analysed.

#### Spinning disk measurements

Spinning disk measurements were performed using the 25 mm-sand blasted Smart Swap geometry. Prior to analysis, colony-biofilms were compressed until an axial force of 0.01 N was detected to normalise the measurements, followed by a 15 s equilibration period. 4 colony-biofilms were analysed at each timepoint.

Stress sweeps were performed by incrementing the shear stress from 0.01–100 Pa at an oscillation frequency of 1 Hz. The point where the storage modulus (G’) intersected with the loss modulus (G”) (See Supplemental Methods), was taken to be an indictor for the yield stress (σ_*y*_)^30^.

Creep-recovery measurements were performed by applying a shear stress of 0.5 Pa for 60 s. A stress of 0.5 Pa was determined to be within the linear viscoelastic region for all *P*. *aeruginosa* colony-biofilms (See Supplementary Methods, Fig. S5). The shear stress was then reduced to 0 Pa and the recovery measured for 120 s. The resulting strain or deformation to the biofilm was monitored as a function of time. The viscosity (η) of the biofilm was determined by dividing the shear stress by the slope of the linear viscous region (R^2^ > 0.95, Supplementary Fig. S4) (η = σ /*slope*). The shear modulus (*G*) was determined by dividing the shear stress by the elastic recovery (*G* = σ / Δγ). The elastic recovery is the initial vertical drop of the recovery portion of the curve (Supplementary Fig. S4).

Frequency sweeps were performed by incrementing the oscillating frequency from 0.1–100 rad/s at a constant stress of 0.5 Pa. The MCI and CCI were calculated using the relationship between the complex modulus (G*) and tanδ (See Supplementary Methods) from *in vitro* models of clearance previously described^22,23,55^. The MCI was calculated at an angular frequency of 1 rad/s and CCI at 100 rad/s.

2 $${\rm{MCI}}=1.62-(0.22\times \,\mathrm{log}\,{G}_{1}^{\ast })-(0.77\times tan{\delta }_{1})$$

3 $${\rm{CCI}}=3.44-(1.07\times \,\mathrm{log}\,{G}_{100}^{\ast })+(0.89\times tan{\delta }_{100})$$

### Statistical analysis

Data are presented as mean ± SD. To test that the data sets conformed to a normal distribution we ran a Shapiro-Wilk test (MedCalc Statistical Software version 17.11.5, Ostend, Belgium). Data was considered to be normal using a p-value > 0.05. All of the data sets were normal except for the Young’s modulus, MCI and CCI of 2-d and 4-d mucoid colony-biofilms. For these data sets means were compared using the non-parametric Kruskal-Wallis test with a Dunn post-hoc test. All other comparisons were made using a One-way ANOVA with a Tukey’s post-hoc test and Student’s t-test. Analyses were performed using GraphPad Prism v.5 (Graphpad Software). Statistical significance was determined using a p-value < 0.05.

### Data Availability

All data generated or analysed during this study are included in this published article (and its Supplementary Information files).

## Electronic supplementary material

Supplementary Information
