# Author Correction: Viscoelastic properties of *Pseudomonas aeruginosa* variant biofilms

**DOI:** 10.1038/s41598-022-12599-2

**Published:** 2022-05-18

**Authors:** Erin S. Gloag, Guy K. German, Paul Stoodley, Daniel J. Wozniak

**Affiliations:** 1 Department of Microbial Infection and Immunity, Microbiology, The Ohio State University, Columbus, OH 43210 USA; 2 Department of Biomedical Engineering, Binghamton University, Binghamton, NY 13902 USA; 3 Department of Orthopedics, The Ohio State University, Columbus, OH 43210 USA; 4 National Centre for Advanced Tribology at Southampton, University of Southampton, Southampton, SO17 1BJ UK

Correction to: *Scientific Reports* https://doi.org/10.1038/s41598-018-28009-5, published online 26 June 2018

This Article contains errors due to an incorrect calculation in the data set to calculate the cough clearance (CCI).

As a result, in the Abstract,

“Theoretical indices of mucociliary and cough clearance predict that mature 6-d parental and RSCV biofilms may show reduced cough clearance from the lung, while early mucoid biofilms may show reduced clearance by both mechanisms.”

should read:

“Theoretical indices of mucociliary and cough clearance predict that RSCV and mucoid biofilms may have altered clearance from the lung, compared to parent biofilms.”

Consequently, Figure 7B contains errors. The correct Figure 7 and its corrected accompanying legend appear below.

**Figure 7:**
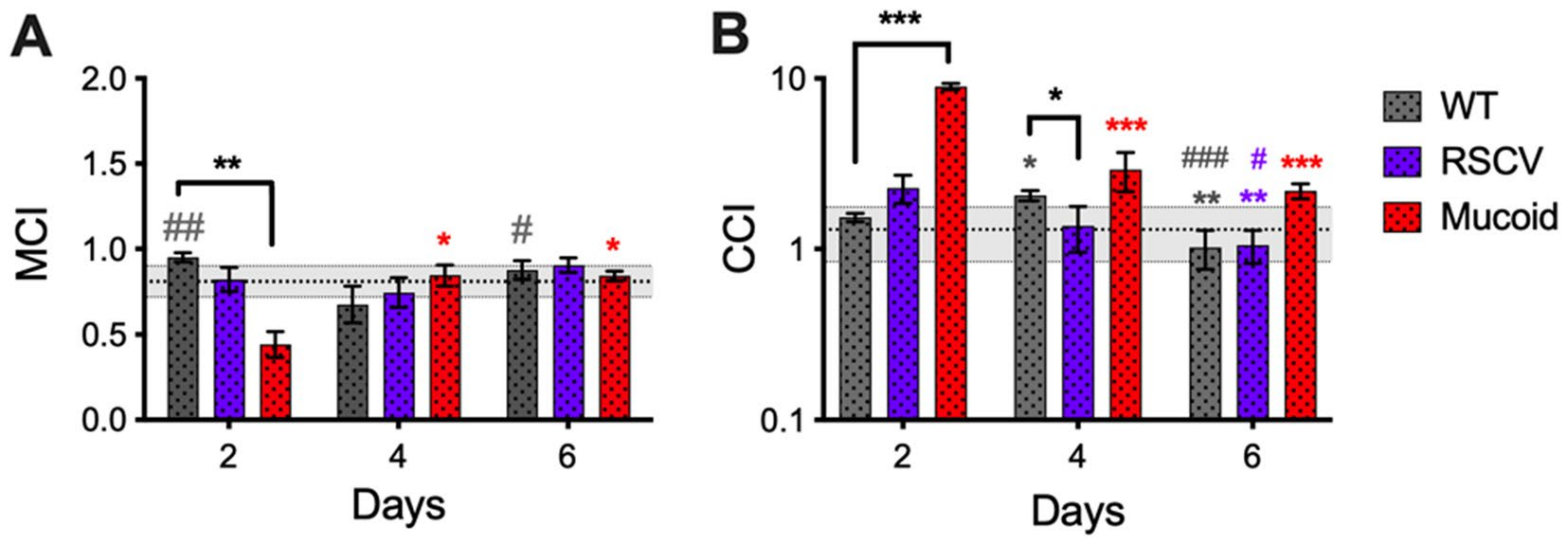
Mucoid colony-biofilms show reduced mucociliary and cough clearance indices. (**a**) The MCI and (**b**) the CCI of *P*. *aeruginosa* colony-biofilms was determined according to equations (2) and (3). The lines at (**a**) 0.81 ± 0.09 and (**b**) 1.3 ± 0.46 indicate the MCI and CCI of CF sputum respectively determined^31^. Data presented as mean ± SD, n = 4. Black * indicates comparisons depicted by the line. Coloured * indicates comparisons to 2-d of the given biofilm. *p-value < 0.05, **p-value < 0.01, ***p-value < 0.001, Coloured ^#^ indicates comparison to 4-d of the given biofilm. ^#^p-value < 0.05, ^##^p-value < 0.01, ^###^p-value < 0.001.

In addition, in the Results section under the subheading ‘Correlation of colony-biofilm viscoelasticity to theoretical mucociliary and cough clearance indices’,

“The fluid behaviour of mucoid biofilms on 2-d correlated to a reduced MCI and CCI compared to WT (Fig. 7). Development of partial elastic-solid behaviour of mucoid biofilms on 4-d and 6-d resulted in an increased MCI and CCI compared to 2-d (Fig. 7).“

should read:

“The fluid behaviour of mucoid biofilms on 2-d correlated to a reduced MCI and increased CCI compared to WT (Fig. 7). Development of partial elastic-solid behaviour of mucoid biofilms on 4-d and 6-d resulted in an increased MCI and reduced CCI compared to 2-d (Fig. 7).“

Lastly, in the Discussion section,

“Early mucoid biofilms, or single mucoid populations may contribute to the inhibition of both mucus clearance mechanisms in CF lungs, as predicted by the low MCI and CCI (Fig. 7). However emergence of non-mucoid populations and the development of partial elastic behaviour at later timepoints resulted in increased MCI and CCI (Fig. 7). Interestingly, the cohesiveness of mucoid biofilms did not change over time, despite these populations (Fig. 3e). Compared to healthy mucus, CF mucus has a greater adhesivity which impairs cough clearance^47^. Therefore, despite the higher MCI and CCI of mucoid biofilms at these later timepoints the sticky mucoid EPS may still contribute to the mucus and reduce clearance.”

should read:

“Early mucoid biofilms, or single mucoid populations may further comprise mucociliary clearance, as predicted by the low MCI (Fig. 7A). However, these populations may be correlated to increased clearance by cough mechanism (Fig 7B). Emergence of non-mucoid populations and the development of partial elastic behaviour at later timepoints resulted in increased MCI and reduced CCI (Fig. 7). Interestingly, the cohesiveness of mucoid biofilms did not change over time, despite these populations (Fig. 3e). Compared to healthy mucus, CF mucus has a greater adhesivity which impairs cough clearance^47^. Therefore, despite the higher MCI and CCI of mucoid biofilms the sticky mucoid EPS may still contribute to the mucus and reduce clearance.”

