# Supplementary material for: Viscoelastic properties of Pseudomonas aeruginosa variant biofilms
Source: Sci Rep. 2018 Jun 26;8:9691. doi: 10.1038/s41598-018-28009-5 (PMC6018706; doi:10.1038/s41598-018-28009-5)
Supplement: Supplementary file 1 — Supplementary Information [file 41598_2018_28009_MOESM1_ESM.docx]

**Viscoelastic properties of *Pseudomonas aeruginosa* variant biofilms**

**Supplemental Information**

Erin S. Gloag, Guy K. German, Paul Stoodley, Daniel J. Wozniak

**Supplementary Materials and Methods**

**Colony-biofilms**

It has been previously reported that bacterial colony-biofilms grown on filter membranes share similar phenotypes to more traditional biofilms, such has hydrated biofilms grown under flow conditions ^1,2^. Previous rheological studies have used this methodology due to the ease in culturing and mechanical analysis ^3^ and for these reasons we utilised colony-biofilms in this study.

Overnight LBNS (10g/L tryptone, 5g/L yeast extract) cultures were diluted to OD_600nm_ 0.1 into fresh 10mL LBNS. Diluted cultures were then poured into a sterile 9mm Petri dish. Nitrocellulose filter membranes (25mm, 0.45μm pore size; Milliopore) were sterilised by exposure to UV for 10min each side. Sterile filter membranes were floated on the culture for approximately 1min before being transferred onto a *Pseudomonas* isolation agar (PIA) plate, culture side up. We used standard laboratory media to keep our study consistent with previous biofilm rheological studies so that our findings could be translatable ^4-9^. The PIA plate was dried for 24h to allow surface moisture to evaporate. We observed that this inoculation method resulted in a reproducibly even coating of the filter, compared to pipetting the culture as per previous reports ^1-3^. The filter was allowed to air dry before being incubated in a humidified chamber at 37°C. Every 24h the filter membrane was transferred to a new PIA plate using sterile forceps.

**Rheology theory**

For spinning disk measurements (excluding creep-recovery measurements) viscoelastic parameters of the biofilm are measured as stress, which is separated into two components depending if the phase angle (δ) is in-phase or out-of-phase with the oscillation. The storage modulus (G’) is the component that is in-phase and describes the elasticity of the material, or the energy that is stored by the material. The loss modulus (G”) is the component that is out-of-phase and describes the viscosity of the material, or the energy that is lost. The storage (G’) and loss (G”) moduli are described by Supplementary equations (S1) and (S2) respectively ^10^:

$$G^{'}= \frac{\sigma_{0}}{\gamma_{0}} cos\delta(S1)$$

$$G" = \frac{\sigma_{0}}{\gamma_{0}} sin\delta(S2)$$

where σ_o_ is the stress amplitude, γ_o_ is the strain amplitude and δ is the phase angle. For ideal elastic materials δ=0, that is completely in-phase. For ideal viscous materials δ=90, that is the material deformation is 90° out-of-phase. For viscoelastic materials both the in-phase and out-of-phase contributions can be resolved and reported as storage (G’) and loss (G”) moduli. The relationship between these moduli for a material can be reported in terms of the ratio of the loss modulus (G”) to the storage modulus (G’) (or the tangent to the phase angle; tanδ) and the complex modulus (G*) which are described by Supplementary equations (S3) and (S4) respectively ^10^:

$$tan\delta= \frac{G"}{G'} (S3)$$

$$G^{*}=G^{'}+ iG" (S4)$$

where *i* is $\sqrt{-1}$.

**Rheometry analysis**

Rheometer apparatus

A TA Instruments Discovery Hybrid Rheometer-2 (HR-2) with the Peltier plate connected to a heat exchanger (TA Instruments) for environmental control was used for all rheological measurements. Where specified the rheometer was fitted with either a 8mm or 25mm-sand blasted Smart Swap parallel plate geometry. For rheological measurements the Peltier plate was covered with a moist Kimwipe to prevent the colony-biofilms from dehydrating during testing. Rheology measurements were performed at 25°C. TRIOS v4 (Discovery HR TA instruments) software was used.

Spinning disk measurements

Strain sweeps were performed to determine the linear viscoelastic region (LVR) and a stress (σ) value that was within this region across all colony-biofilms (Supplementary Fig. S5). Using values within the LVR permits analysis of the biofilm without disruption to the mechanical integrity of the biofilm. Strain sweep measurements were performed by incrementing the strain (γ) from 0.01-100% at an oscillation frequency of 1Hz. The LVR was determined for each biofilm (Supplementary Fig. S5A; dotted black lines). A stress (σ) value of 0.5Pa was determined to be within the LVR for all colony-biofilms (Supplementary Fig. S5B; dotted black line). A stress (σ) of 0.5Pa was also determined to be within the LVR from stress sweeps (Supplementary Fig. S1). This value was used in subsequent tests.

**Lawn-biofilms and rheological analysis**

Overnight LBNS cultures was diluted to OD_600nm_ 0.1 and 200μL was spread plated onto PIA plates and incubated 24h at 37°C. Biofilm was then scraped off the agar and transferred directly to the Peltier plate of the rheometer.

For rheological analysis biomass from 5 plates for WT and RSCV were combined for each replicate in order to have enough sample for analysis. Enough biomass of mucoid lawn-biofilms was obtained from 1 plate. Rheological analyses were performed as previously described with the exception that the trimming gap was set to 500μm and that stress sweeps were performed by incrementing the stress (σ) from 0.01-1000Pa.

**Supplementary Results and Discussion**

**Scraped *P. aeruginosa* lawn-biofilms have different rheological properties compared to filter colony-biofilms**

It has previously been identified that RSCV scraped lawn-biofilms were tougher and more elastic compared to the parent strain ^5^. This is contrary to what was observed here, where the biophysical properties of WT and RSCV colony-biofilms were relatively similar. To determine if these differences were due to the analysis of different biofilm types, scraped lawn-biofilms versus colony-biofilms, we performed stress sweeps (Supplementary Fig. S2A) and frequency sweeps (Supplementary Fig. S3) on 24h WT, RSCV and mucoid scraped lawn-biofilms.

The yield stress (σ_y_) of the scraped lawn-biofilms was determined from stress sweeps, as for the colony-biofilms. This revealed that the scraped lawn-biofilms were significantly stronger than their colony-biofilm counterparts for all three strains, with each showing a greater yield stress (Fig. 4, Supplementary Fig. S2B). However, despite this the yield stress of WT and RSCV scraped lawn-biofilms were similar (Supplementary Fig. S2B). Furthermore, the yield stress of mucoid scraped lawn-biofilms was similar to WT (Supplementary Fig. S2B), which was not observed for the colony-biofilms (Fig. 4).

Likewise the viscoelastic profiles of scraped lawn-biofilms (Supplementary Fig. S3) were different compared to their colony-biofilm counterparts (Fig. 6). While displaying gel properties, both WT and RSCV scraped lawn-biofilms showed more elastic dominant behaviour, with the storage modulus (G’) plateau greater in magnitude than that for the colony-biofilms (Fig. 6, Supplementary Fig. S3). Mucoid scraped lawn-biofilms displayed viscoelastic crossover behaviour (Supplementary Fig. S3), similar to 4-d and 6-d colony-biofilms (Fig. 6C). However, the storage modulus (G’) at higher frequencies was greater than that for 4-d and 6-d colony-biofilms (Fig. 6C, Supplementary Fig. S3).

Together this indicates that *P. aeruginosa* scraped lawn-biofilms and colony-biofilms display different rheological properties. We predict this is due to scraping disrupting the structure of the biofilm, inducing altered or artificial biophysical behaviour. *P. aeruginosa* colony-biofilms show stratification of protein synthesis ^2^. It can be assumed that stratification of the EPS within these biofilms would also occur. This would result in the formation of slip-like planes during rheology measurements. Assuming this stratification similarly occurs in lawn-biofilms, scraping would result in coalescence of these layers. This may account for the observation here that the scraped lawn-biofilms were more elastic-solid compared to the colony-biofilms. Despite these differences, WT and RSCV scraped lawn-biofilms still displayed relatively similar biophysical properties. Therefore the different modes of biofilm growth do not account for the differences observed here, to that reported by Kovach *et al* ^5^ for RSCV and WT biofilms. We predict this may be due to different media used, as here biofilms were grown on PIA whereas Kovach *et al* utilised LB^5^.

**RSCV colony-biofilms as drying colloidal films can account for the wrinkled phenotype and rheological properties**

A striking feature of RSCV colonies are the wrinkled surfaces they form ^11,12^. This morphology was also observed for the RSCV colony-biofilms (Fig. 1A). We speculate that RSCV colony-biofilms may behave similar to drying colloidal films. For these materials, drying induces the formation of a viscoelastic shell as particles aggregate at the air-surface interface. Capillary forces at this interface act on the shell driving its deformation as it responds by viscous behaviour. At a critical threshold, where the capillary forces exceed the internal stabilising forces, the shell transitions from a viscous liquid to a elastic solid and buckles ^13,14^. This results in the dissipation of in-plane stresses and relaxation of the material ^15^. Considering the wrinkled network of RSCV colony-biofilms as an elastic skin encasing a viscoelastic liquid core accounts for the mechanical behaviour observed here. Specifically, the bulk rheological properties of RSCV biofilms did not change significantly over time and were relatively similar to WT.

Wrinkle formation may therefore be a mechanism bacterial communities employ to release stresses. Whether biofilms undergo wrinkle formation *in vivo* and if these structures play a role in similarly releasing stresses, or are just an interesting phenomenal artifact that occurs when these colonies are grown at an air interface is yet to be determined.

**Supplementary References**

1 Anderl, J. N., Franklin, M. J. & Stewart, P. S. Role of antibiotic penetration limitation in *Klebsiella pneumoniae* biofilm resistance to ampicillin and ciprofloxacin. *Antimicrobial agents and chemotherapy* **44**, 1818-1824 (2000).

2 Walters, M. C., Roe, F., Bugnicourt, A., Franklin, M. J. & Stewart, P. S. Contributions of antibiotic penetration, oxygen limitation, and low metabolic activity to tolerance of Pseudomonas aeruginosa biofilms to ciprofloxacin and tobramycin. *Antimicrobial agents and chemotherapy* **47**, 317-323 (2003).

3 Jones, W. L., Sutton, M. P., McKittrick, L. & Stewart, P. S. Chemical and antimicrobial treatments change the viscoelastic properties of bacterial biofilms. *Biofouling* **27**, 207-215, doi:10.1080/08927014.2011.554977 (2011).

4 Korstgens, V., Flemming, H. C., Wingender, J. & Borchard, W. Uniaxial compression measurement device for investigation of the mechanical stability of biofilms. *Journal of microbiological methods* **46**, 9-17 (2001).

5 Kovach, K. *et al.* Evolutionary adaptations of biofilms infecting cystic fibrosis lungs promote mechanical toughness by adjusting polysaccharide production. *npj Biofilms and Microbiomes* **3**, 1 (2017).

6 Rmaile, A. *et al.* Microbial tribology and disruption of dental plaque bacterial biofilms. *Wear* **306**, 276-284 (2013).

7 Stoodley, P., Lewandowski, Z., Boyle, J. D. & Lappin-Scott, H. M. Structural deformation of bacterial biofilms caused by short-term fluctuations in fluid shear: an in situ investigation of biofilm rheology. *Biotechnology and bioengineering* **65**, 83-92 (1999).

8 Vinogradov, A., Winston, M., Rupp, C. & Stoodley, P. Rheology of biofilms formed from the dental plaque pathogen Streptococcus mutans. *Biofilms* **1**, 49-56 (2004).

9 Wloka, M., Rehage, H., Flemming, H.-C. & Wingender, J. Structure and rheological behaviour of the extracellular polymeric substance network of mucoid Pseudomonas aeruginosa biofilms. *Biofilms* **2**, 275-283 (2005).

10 Ferry, J. D. *Viscoelastic properties of polymers*. (John Wiley & Sons, 1980).

11 Kirisits, M. J., Prost, L., Starkey, M. & Parsek, M. R. Characterization of colony morphology variants isolated from Pseudomonas aeruginosa biofilms. *Applied and environmental microbiology* **71**, 4809-4821, doi:10.1128/aem.71.8.4809-4821.2005 (2005).

12 Starkey, M. *et al.* Pseudomonas aeruginosa rugose small-colony variants have adaptations that likely promote persistence in the cystic fibrosis lung. *Journal of bacteriology* **191**, 3492-3503, doi:10.1128/jb.00119-09 (2009).

13 Dufresne, E. R. *et al.* Flow and fracture in drying nanoparticle suspensions. *Physical review letters* **91**, 224501, doi:10.1103/PhysRevLett.91.224501 (2003).

14 Tsapis, N. *et al.* Onset of buckling in drying droplets of colloidal suspensions. *Physical review letters* **94**, 018302 (2005).

15 Routh, A. F. Drying of thin colloidal films. *Reports on Progress in Physics* **76**, 046603 (2013).

**Supplementary Figures**

**
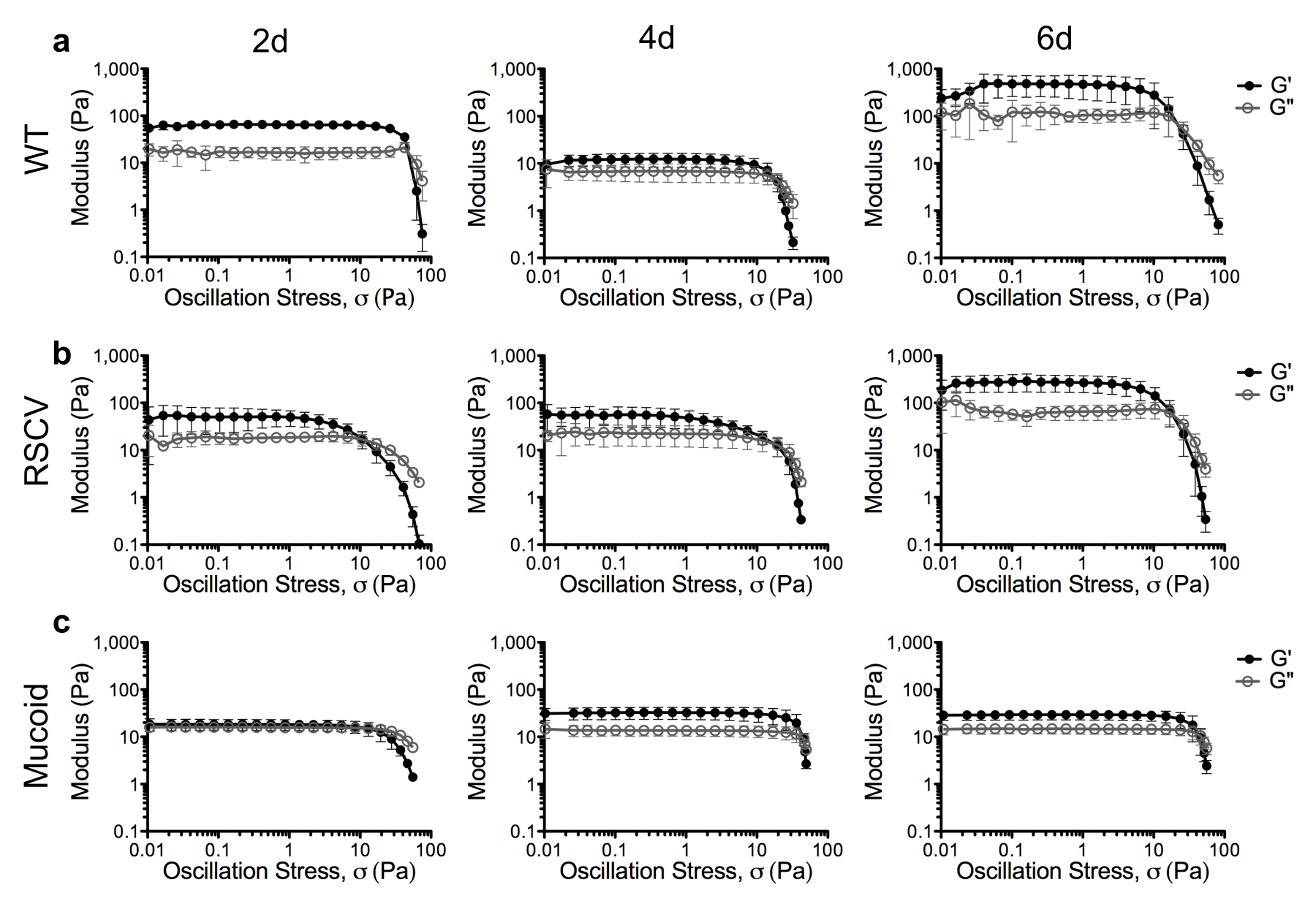
**

**Supplementary Figure 1: Stress sweeps of *P. aeruginosa* colony biofilms.** Stress sweeps were performed on **(a)** WT, **(b)** RSCV and **(c)** mucoid colony biofilms at 2-, 4- and 6-d (labelled) by incrementing the stress (σ) from 0.01-100Pa. Data presented as mean ± SD; n=4.

**
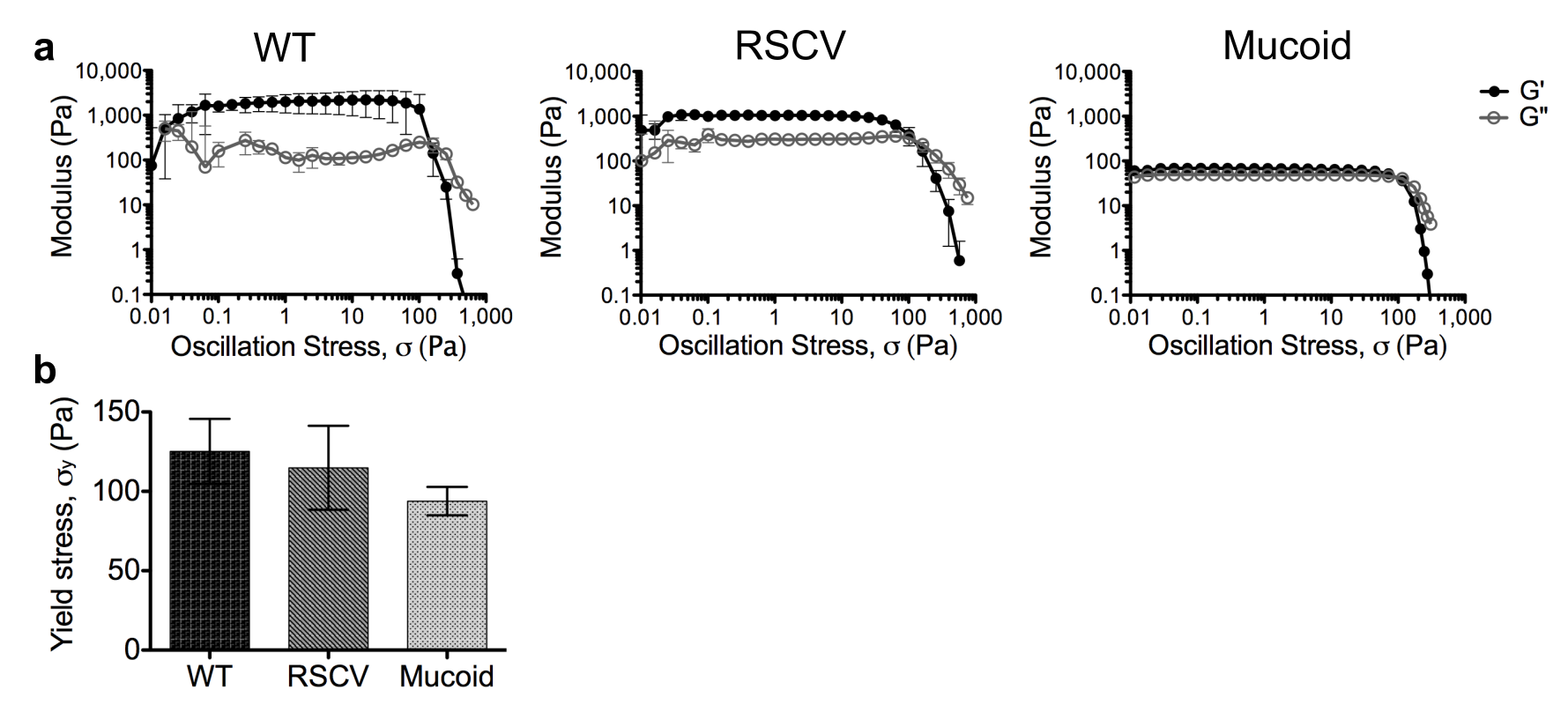
**

**Supplementary Figure 2: *P. aeruginosa* lawn-biofilms have a higher yield stress compared to colony-biofilms. (a)** Stress sweeps were performed on *P. aeruginosa* WT, RSCV and mucoid (labelled) scraped lawn biofilms by incrementing the stress (σ) from 0.01-1000Pa. **(b)** Yield stress (σ_y_) of *P. aeruginosa* lawn biofilms. The yield stress (σ_y_) was taken to be the point of viscoelastic crossover where the storage modulus (G’) and the loss modulus (G”) intersected. Data presented as mean ± SD, n =4.

**
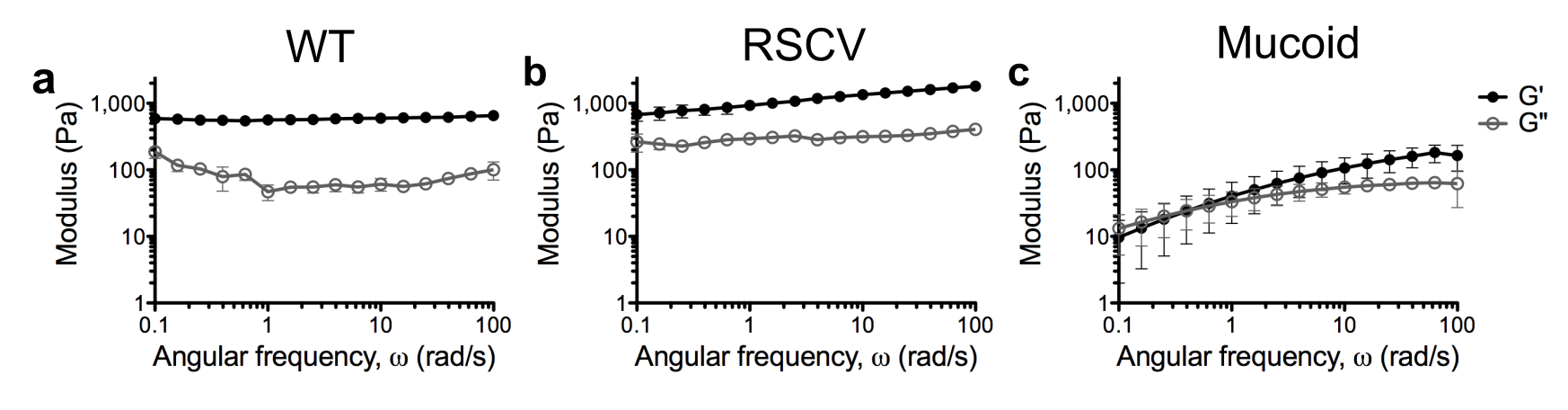
**

**Supplementary Figure 3: *P. aeruginosa* lawn-biofilms have a more elastic dominate behaviour compared to colony-biofilms.** Frequency sweeps were performed on *P. aeruginosa* **(a)** WT, **(b)** RSCV and **(c)** mucoid (labelled) scraped lawn-biofilms by incrementing the angular frequency (ω) from 0.1-100rad/s. Data presented as mean ± SD; n=4.


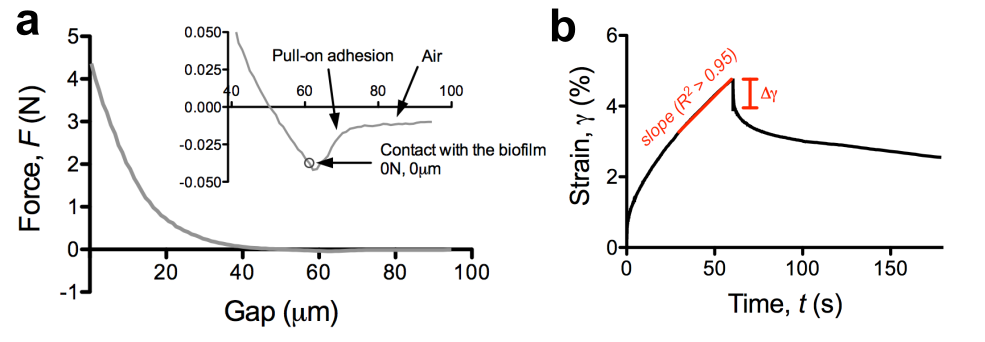


**Supplementary Figure 4: Data analysis of *P. aeruginosa* colony-biofilms.** **(a)** Example of raw data from uniaxial compression measurement of WT 2-d colony-biofilm. Inset depicts the pull-on adhesion of the geometry as at approached the biofilm surface. The point where the force (*F*) began to increase after this point was taken to be the top of the biofilm. The gap and force measurements were normalised to this point. **(b)** Example of creep-recovery data of WT 2-d colony-biofilm. Where the slope and the elastic recovery measurements were taken are labelled on the graph. The slope of the linear viscous response was used to calculate the viscosity. The linear viscous response typically occurred at the end of the creep portion of the curve. The response was determined to be linear where R^2^ > 0.95. As indicated on the graph the elastic recovery is the initial vertical drop of the recovery portion of the curve. The length of this recovery response was measured (Δγ) and used to calculate the shear modulus.


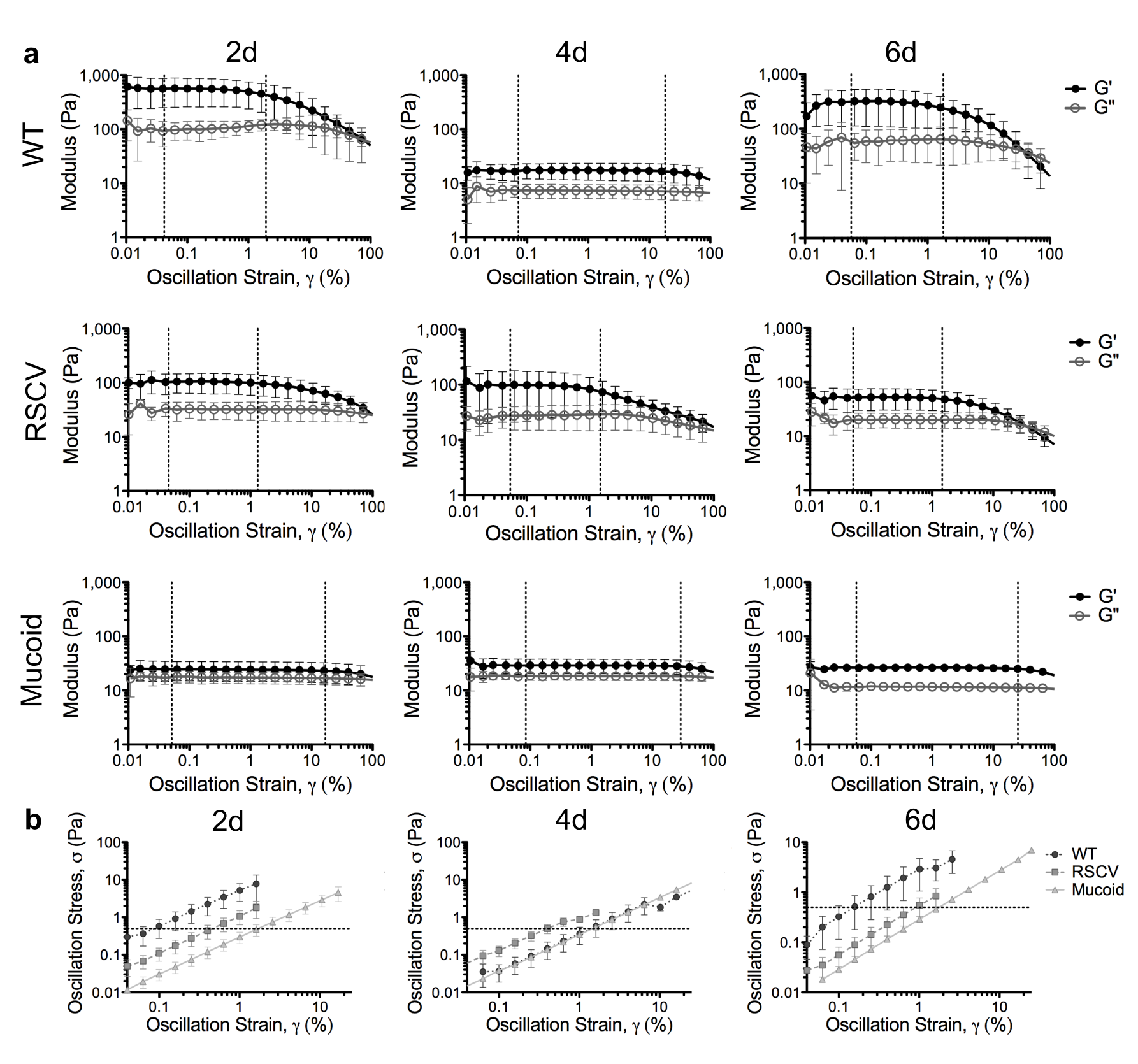


**Supplementary Figure 5: Linear viscoelastic region of *P. aeruginosa* colony biofilms.** **(a)** Strain sweeps were performed on WT, RSCV and mucoid colony biofilms at 2-, 4- and 6-d (labelled) by incrementing the strain (γ) from 0.01 – 100%. Dashed line indicates the average linear viscoelastic region (LVR) for each biofilm. Data presented as mean ± SD; n=4. **(b)** Strain (γ) values from the LVR depicted in **(a)** for each biofilm with the corresponding stress. A stress (σ) of 0.5Pa (dashed line) was identified to be within the LVR for all biofilms. This stress (σ) was used for subsequent measurements.
